# Supplementary material for: Capturing the dynamics of microbial interactions through individual-specific networks
Source: Front Microbiol. 2023 May 15;14:1170391. doi: 10.3389/fmicb.2023.1170391 (PMC10225591; doi:10.3389/fmicb.2023.1170391)
Supplement: Supplementary file 1 [file Data_Sheet_1.pdf]

Supplementary Material

Capturing the Dynamics of Microbial Interactions Through Individual-Specific Networks

Behnam Yousefi†, Federico Melograna†, Gianluca Galazzo, Niels van Best, Monique Mommers, John Penders, Benno Schwikowski, Kristel Van Steen*

*** Correspondence:** Corresponding Author: kristel.vansteen@uliege.be
† Equal Contribution

1 Supplementary Information

Individual Specific Networks

The construction and interpretation of individual-specific networks (ISN) have obtained renewed attention in the context of precision medicine. Here, we define an ISN as a network for which both nodes and edges can be allocated to a single individual. An example of an ISN that only uses data from a single individual could be, for instance, a beta-cell interaction network for beta-cells residing in an individual's tissue sample and edges defined by changes in intracellular calcium concentrations (Gosak et al., 2018). Alternatively, ISNs are constructed from a population of individuals. One procedure to construct such ISNs from reference data was proposed by(Kuijjer, Hsieh, et al., 2019; Kuijjer, Tung, et al., 2019) and has been applied to several scenarios (Jahagirdar & Saccenti, 2020) In (Jahagirdar & Saccenti, 2020) the authors compare and evaluate Kuijjer's LIONESS (Linear Interpolation to Obtain Network Estimates for Single Samples) method (Kuijjer, Tung, et al., 2019) and ssPCC (single sample network based on Pearson correlation) (Liu et al., 2016) for metabolomics measurements and two independent groups of individuals (for instance cases and controls). In contrast, in ssPCC methodology (Liu et al., 2016), only the perturbation that an individual causes to an interaction network, when adding the individual to the population of samples, is considered. In either case, these ISN construction methods require the inference of a network via a pool of individuals or samples (hereafter referred to as a global network), at some point down the line.

Global Network Construction

MAGMA is the primary method for global network construction employed. It consists of several steps. Firstly, microbial abundances are modelled using a ZINB (zero-inflated negative binomial) that can also accept covariates. Furthermore, a maximum likelihood approach is used to infer the ZINB. On top of that, a sparse precision matrix $\tilde{\theta}_\rho$ is estimated. Different values of a penalization parameter ρ yield different precision matrices, and hence ρ is optimized considering three approaches as referred to in Cougoul et al.((Cougoul et al., 2019). In this work, we selected the rotation information criteria (RIC – (Zhao et al., 2020)) as default in the MAGMA package. Finally, the optimized penalization parameter ρ^* is employed to identify the amplicon sequence variants (ASV) network from the non-zero elements $\tilde{\theta}_{\rho^*}$. We refer the interested reader to the work of Cougoul et al. (Cougoul et al., 2019) for an in-depth understanding of the MAGMA algorithm.

An alternative to MAGMA is the widely used SparCC approach. SparCC applies a log transformation of the components and then estimates the Pearson correlations between those

components. The algorithm calculates iteratively a "basis correlation", with the underlying assumption that most pairs do not correlate. This approach reduces the amount of spurious negative correlations derived from the compositional nature of the data (Friedman & Alm, 2012). To amend the problem that zero would cause to the log transformation it employs a pseudo-count, i.e., assigning a small fraction to ASVs not detected in a sample. A fast implementation of SparCC, used in this study, is given by FastSpar.

There are several motivations behind MAGMA's choice over SparCC. MAGMA can include covariates and does not need to adjust via pseudo-counts. Moreover, MAGMA is able to infer direct associations using notions of conditional independence. SparCC may fail to generate a positive definite covariance matrix. Notably, both SparCC and MAGMA work directly on the ASV counts. Hence, no pre-transformation is needed. Moreover, both methods consider the compositional zero-inflated and overdispersed nature of the data.

SparCC Analyses

We have repeated the microbiome dynamic analyses with SparCC as a global network construction method instead of MAGMA. The results are shown in Figure S10. We observe differences with MAGMA for the number and strength of connections, which appear to be more abundant and stronger in SparCC analysis. The lack of natural sparsification in SparCC can explain this. A comparison of the global MAGMA and SparCC network shows the impact the selected network construction method has on the final results. Variation in network properties obtained for networks via different inference methods has been observed before (Kishore et al., 2020). Aggregated consensus networks, across a variety of construction methods, may be opted for to handle heterogeneous network solutions.

Stability Analysis

As an alternative to borrowing strengths from multiple global network inference methods, we investigated the stability of the selected inference method. In particular, we performed bootstrap-based stability checks for inferred discovery MAGMA networks, separately for the two time points considered in this study (months 6m and 9m after birth). MAGMA's population networks at 6m (and 9m) had 95 nodes. The mean edge weight was $-1.98 * 10^{-3}$ ($-2.01*10^{-3}$); about 5% of possible edges were inferred to be present (184 (216) out of 4465 possible node pairs). For both populations of individuals (i.e., corresponding to 6m and 9m measurements), 1,000 resampling were obtained with replacement. The replicate datasets, of equal population sizes as the originals were used to reconstruct MAGMA networks.

Non-parametric bootstrap was carried out with the *bootnet* R package version 1.4.3 (Epskamp et al., 2018), which allows estimating the stability of edge weights. Bootstrapped estimates of edge weights, with their 95% confidence intervals (CI), for 4,465 edges, are displayed in Figure S11 (A 6m, B 6m). At 95% confidence, the minimum lower bound over all 4,465 edges was -0.47 (-0.48) and the maximum upper bound was 0.15 (0.13) at month 6m (9m). The highest instability for edge weights (i.e. bootstrap estimates with highest standard deviation) was observed for the microbial pairs *Lachnospiraceae* (Family, no lower classification available) and *Coprococcus* sp. at 6m, and *Bifidobacterium sp.* and TM7-3 (Class, no lower classification) at 9m. None of these were highlighted in downstream analyses. For all MAGMA inferred zero-weight edges in the discovery networks (4,281 and 4,249, at 6m and 9m, respectively), the bootstrap confidence intervals contained zero. Hence, we can be quite confident about the absence of edges in the discovery MAGMA networks.

The threshold networks based on 2.5% - 97.5% bootstrapped quantiles for each edge, obtained via the R function *BootThreshold* (operating on *bootnet* output) are displayed in Figure S12A, B. These sparse bootstrap threshold networks, for which an edge is only present when zero is not included in the 2.5% - 97.5% bootstrap interval, only had 12 and 8 edges present (non-zero edge weights) at 6m and 9m, respectively. This implies that for quite a number of edges, with non-zero weights in the discovery networks (about 5% of the total number of edges, see before), the corresponding 95% bootstrap CI contained zero. These results seem to indicate that a larger amount of uncertainty exists around the non-zero inferred edge weights, compared to zero inferred weights, in the discovery networks. However, the bootstrap threshold networks do not take into account that zero may be at the boundary of bootstrap intervals. Hence, we also considered an alternative way to construct networks from bootstrap replicates. In particular, we used the *bootInclude* function of the *bootstrap* package to build inclusion networks by considering only edges present in more than half (50%) of the replicate MAGMA networks. For these inclusion networks, on average, 94.2 % and 95.7% of all possible edges had zero weights for m6 and 9m, respectively. The inclusion networks at 6m and 9m had 118 and 82 edges, respectively, suggesting that in our study the discovery MAGMA networks may be liberal in the edges. The displays of the inclusion networks (Figure S12C, D) show solid modularity and a giant component, i.e., a connected component of appreciable size, in terms of number of nodes. We took the biggest connected component in threshold, inclusion and discovery network and compare it which each other. The size of the giant component, in terms of the number of nodes, is as follow: at 6m (9m): 4 (2), 79 (50), and 85 (91) for threshold, inclusion and discovery networks, respectively. The number of edges between the nodes in these giant components were quite different across networks: at 6m (9m) 3 (1), 112 (68), and 182 (216) for threshold, inclusion and discovery networks, respectively. The larger agreement between inclusion and discovery networks is also apparent from Figure S13.

For completion, we further compared discovery and inclusion network nodes in terms of their node strength (i.e. the sum of all edge weights for a node) and relative ranking. We took all 95 network nodes (hence, no longer focusing on the giant component discussed before). We used Kendall's rank correlation *tau* as a test statistic, which tests the degree of similarity between two sets of ranks. The hypothesis of independence of the ranking was rejected at 6m (*tau* = 0.75, p-value < 2.2e-16) and at m9 (*tau* 0.78, p-value < 2.2e-16). See also Tables SII and SIII. For each network, we then focused on the top 10 nodes with the highest node strengths and investigated the overlap between inclusion and threshold networks (Figure S14). For time point 6m, only *Bifidobacterium bifidum* and *Enterobacteriaceae* (Family, no available lower classification) were shared, and for time point 9m, the networks only shared *TM7-3* (Class, no available lower classification). The overlap between inclusion and discovery networks for corresponding top 10 nodes, was as follows: m6: 7 nodes and m9: 6 nodes. Focusing on common edges between the discovery networks, inclusion and threshold networks, at each time point, we found 12 edges at 6m and 8 edges at 9m, as illustrated in Figure S15A, B.

The population size may also affect the stability of population-based inferred networks. To investigate this in greater depth, we implemented 1,000 case-dropping bootstraps using the *bootnet* R package that we also used before (Epskamp et al., 2018). Case-dropping bootstrap is a variant of the bootstrap algorithm. Instead of sampling the data with replacement, samples are generated by randomly dropping a certain number of samples from the original dataset. The remaining samples are then used to estimate a desired parameter. The approach thus allows assessing the sensitivity of parameter estimation to changing samples. Here, we explored the stability of edge weights and node strengths. We progressively excluded 10% - 70% of the total population (in steps of 10%). The analyses focused on computing and interpreting the correlation stability (CS) coefficient, defined as the mean percentage of our sample that can be dropped to maintain correlation values ≥ 0.7 on 95% of the bootstrap repetitions. The 1,000 correlation values measure the association between the initially observed statistics (i.e., edge weight, node strength) and the corresponding 1,000 bootstrap

statistics. Epskamp (Epskamp et al., 2018) suggest evidence of stability when CS > 0.25. According to this criterium, edge weights and node strengths were stable at m6 (CS = 0.358 and 0.284, respectively) and at m9 (CS = 0.368 and 0.284, respectively), as showed in Figure S16. On the one hand, high stability may be preferred. On the other hand, high stability may make individual-specific network analysis redundant. Notably, leaving one individual out from a study population to construct an ISN involved removing 1.2% and 1.4% of samples for 6m and 9m networks, respectively.

A customized implementation of repetitive weighted random walks

To obtain the node visit probabilities at the decoder side of our MNDA framework, we developed a *fixed-length weighted random walk algorithm*. Accordingly, the walker is restricted to taking a limited number of steps to a predefined fixed length. This enables us to characterise the local structures of the ISN. More specifically, we start "walks" from each node and compute the probability of visiting all the other nodes during the walk. We tried different values for walker length (≤ 10) to keep the walks local. We finally chose a walk length of 5 that resulted in the smallest mean square error for EDNN. It is worth mentioning that the results were fairly stable across walk lengths. To calculate the node visit probabilities, we repeated the walking process 100 times for each node.

Since the degree of co-occurrences of microbes may be highly variable, we proposed to use a *weighted random walk algorithm* to take the edge weights into account. To move from node i and determine the next step in a simple random walk algorithm, all the neighbouring nodes have the same probability of visiting $P(i \rightarrow j) = \frac{1}{\|\mathcal{N}_i\|}$, where $\|\mathcal{N}_i\|$ is the set of neighbours (distance 1) of node i with operator $\|\cdot\|$ counting its members. In the weighted version of this algorithm, the probability of moving from node i to node j is proportional to the edge weight w_{ij} linking node i to node j:

$$P(i \rightarrow j) = \frac{w_{ij}}{\sum_{j \in \mathcal{N}_i} w_{ij}}$$

Although the idea of weighted random walks is not new, to our knowledge, no customized code was available for their use in our framework. Our code is provided as part of a *GitHub* repository that covers the workflows used in this manuscript (https://github.com/H2020TranSYS/microbiome_dynamics).

A novel implementation of ensemble consensus clustering

Many clustering methods can be more robust by *consensus clustering* (CC). Here, we propose a CC framework to achieve robustness by repeatedly applying MNDA (50 repetitions) and subsequent clustering of microbes based on their cosine distance. As mentioned before, cosine distance is computed across all pairs of possible microbes, spanning data on months 6 and 9. The adopted clustering strategy is *k-means*; the number of clusters k is determined by maximising the *Silhouette index*. For M microbes, of which dynamics need to be tracked across time points, we calculate the $2M \times 2M$ matrix of co-clustering. Matrix entries are the frequencies of a pair of microbes (a single microbe at two time points or two microbes at some point in time) jointly belonging to the same cluster among all clusterings. This co-clustering matrix can be viewed as a robust similarity matrix based on which we can define a robust "distance" between any pair of nodes in the original MNDA embedding space. The more distant the two nodes in the embedding space are, the more their local neighbourhoods differ. Hence, when two nodes refer to the same microbe, measured at different time points, high co-cluster similarity implies low local

neighbourhood dynamics for that particular microbial taxon, considering its global microbial co-occurrence neighbourhoods at months 6 and 9. We implemented consensus clustering proposed by Monti et. al (Monti et al., 2003) on the co-clustering matrix to obtain the optimum number of clusters along with the clustering results. As depicted in Figure S8B, the algorithm suggests two clusters of microbes by calculating the area under the curve of the *empirical cumulative distribution*. For additional details, we refer to Monti et al. (Monti et al., 2003)\.

---

2 Supplementary Figures and Tables

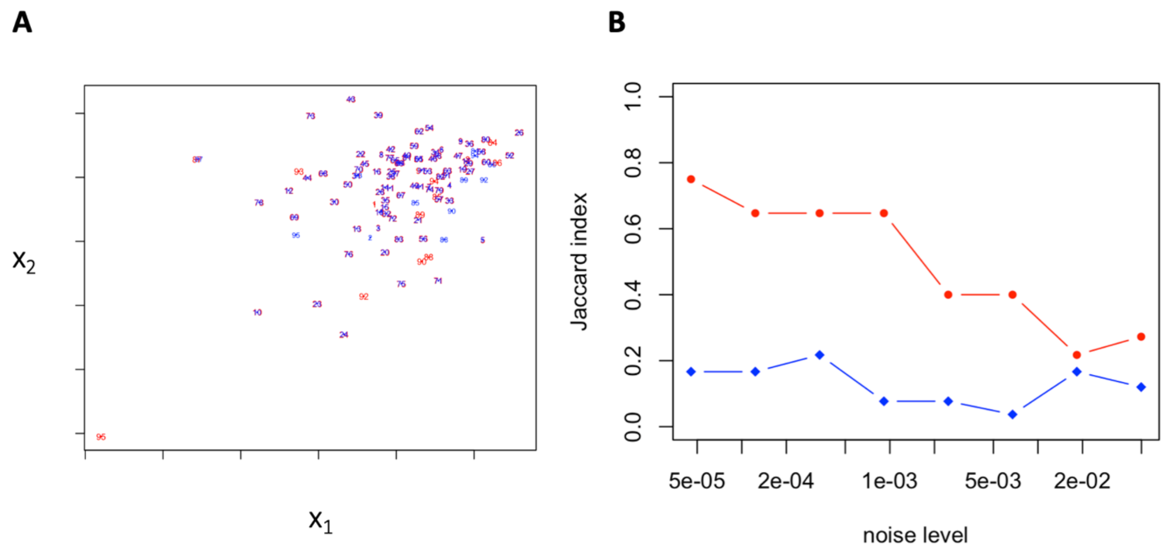

Figure S1: Implementation of MNDA on simulated data. (A) A 2D representation of the EDNN embedding space for a simulated two-layered multiplex network. Each number is a node (red: layer 1 and blue: layer 2). All the nodes whose local neighbourhoods are not changing in the network fall on top of each other. (B) The Jaccard index between the pre-set varying nodes and the inferred varying nodes against the noise increase. The red and blue diagrams correspond to the MNDA-based and eigendecomposition-based methods.

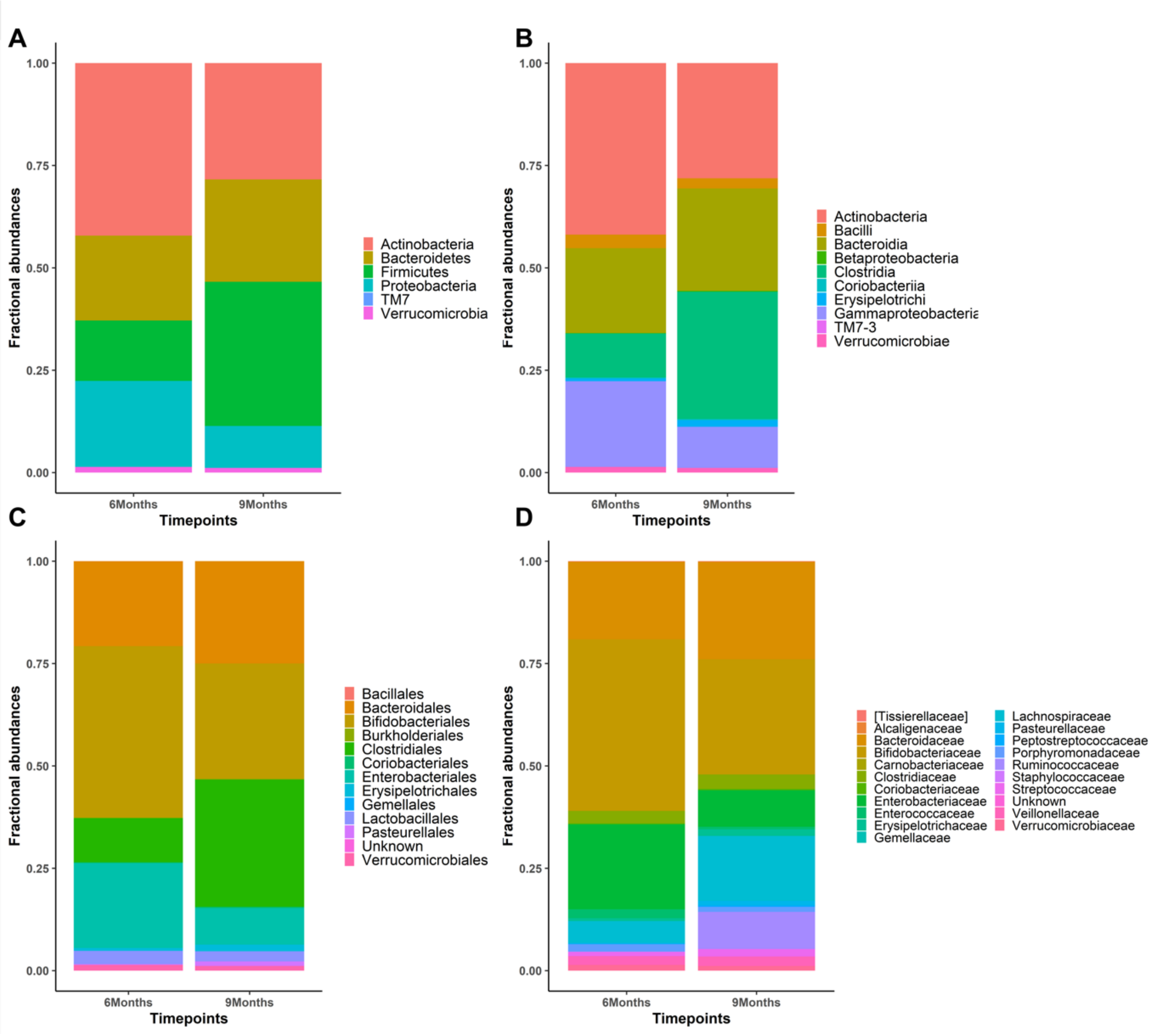

Figure S2: Fraction of relative abundances belonging to each (A) phylum, (B) order, (C) class, (D) family in 6 months and 9 months on the 95 microbes selected with MAGMA pre-processing for the 69 paired newborns.

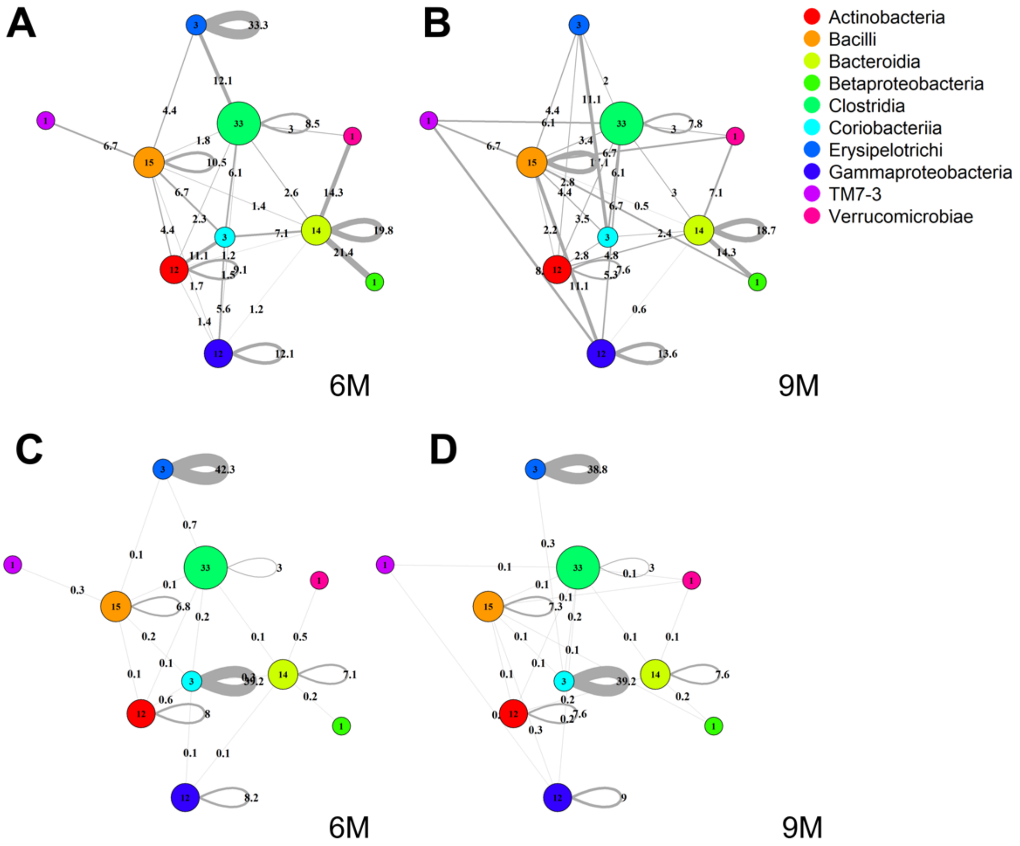

Figure S3: Representation of binary (top) and continuous (bottom) *Class* taxa interaction at months 6 (left) and 9 (right). The binary graphs show an increase in significant interactions between taxa grouped per Class from months 6 to 9. This is not translated into the weighted MAGMA networks.

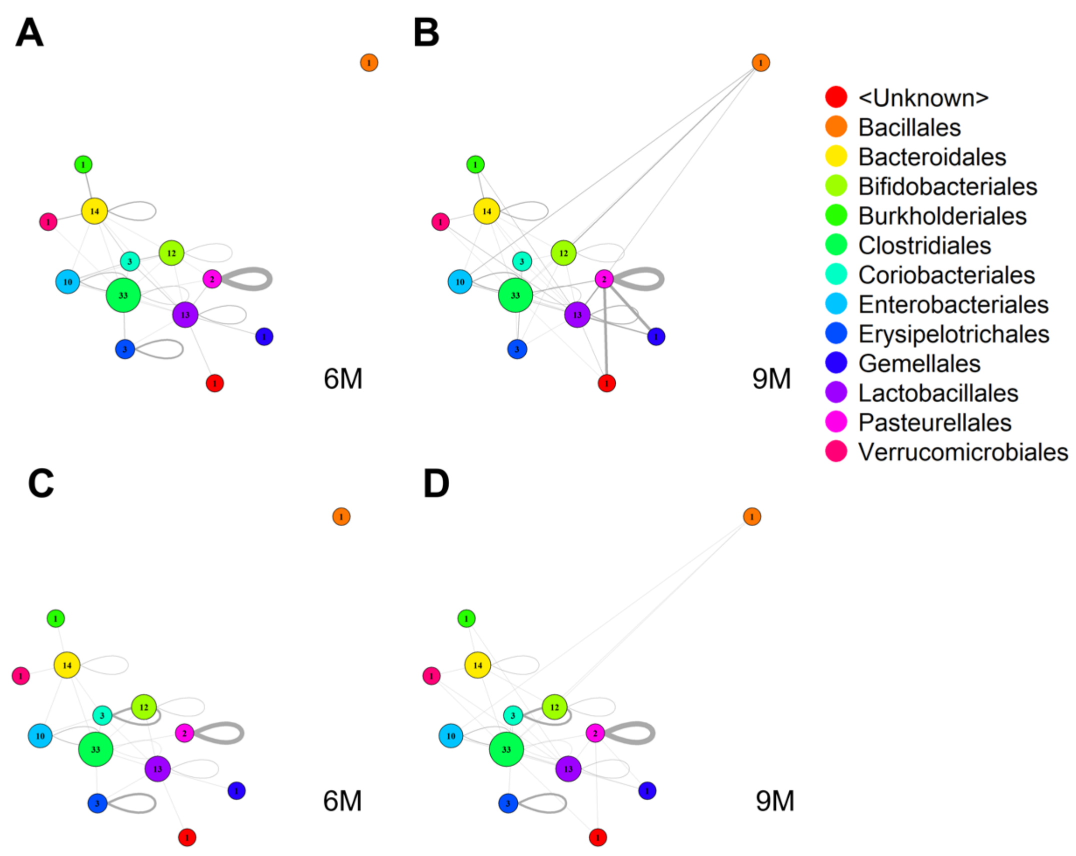

Figure S4: Representation of binary (top) and continuous (bottom) *Order* taxa interaction at months 6 (left) and 9 (right). From the binary graphs, we can see an increase in the significant interactions between taxa grouped per *Order* from months 6 to 9.

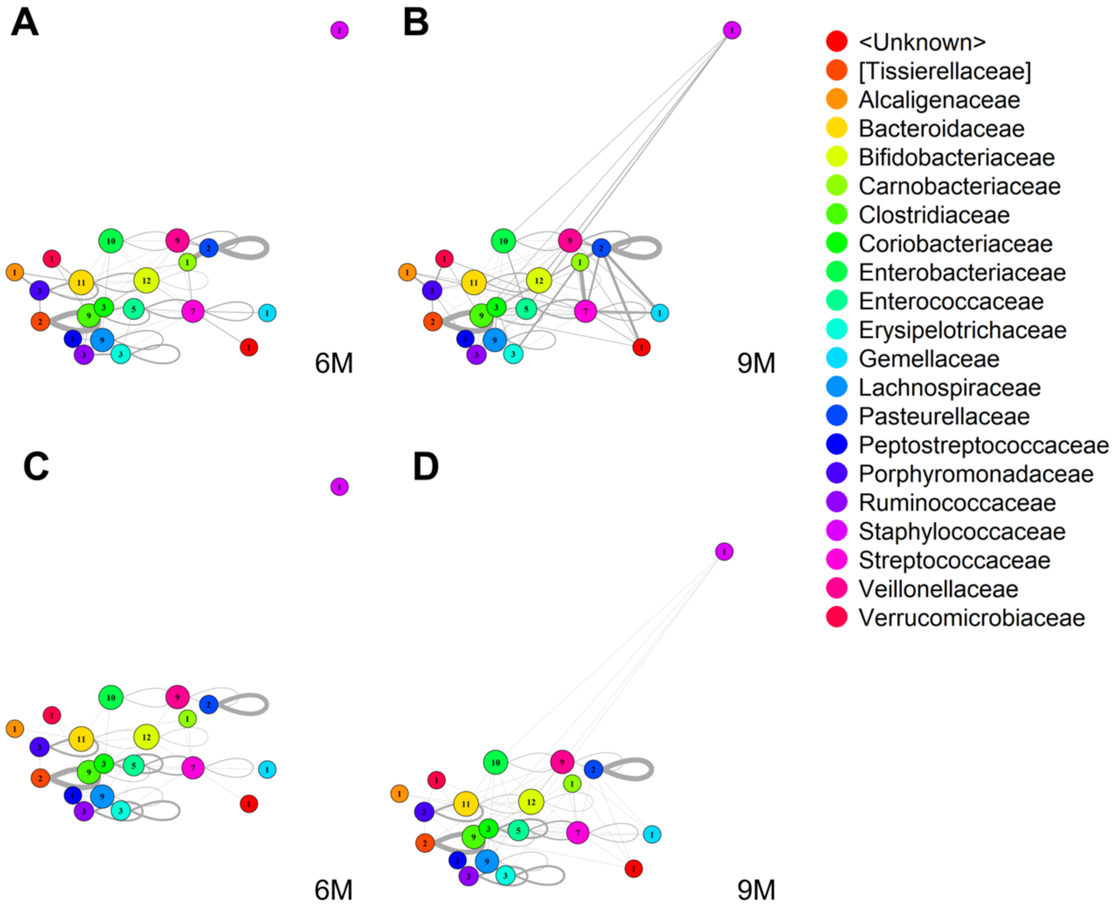

Figure S5: Representation of binary (top) and weighted (bottom) *family* taxa interactions at months 6 (left) and 9 (right). The binary graphs show an increase in significant interactions between taxa grouped per family from months 6 to 9. Edge weights are not shown.

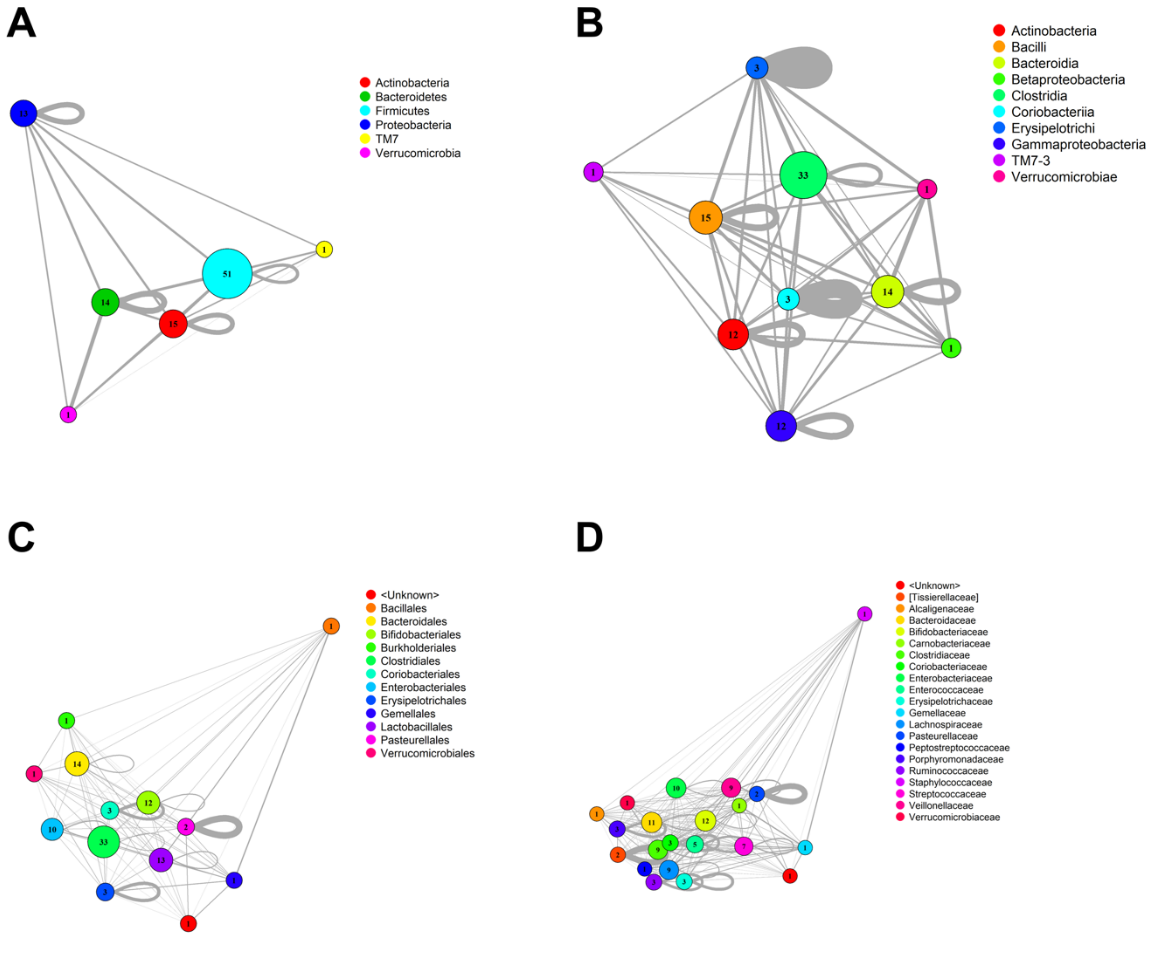

Figure S6: Representation of weighted taxa interactions at month 6, grouped for (A) Phylum, (B) Class, (C) and (D) Families with the SparCC method. SparCC does not downplay edges as much as MAGMA

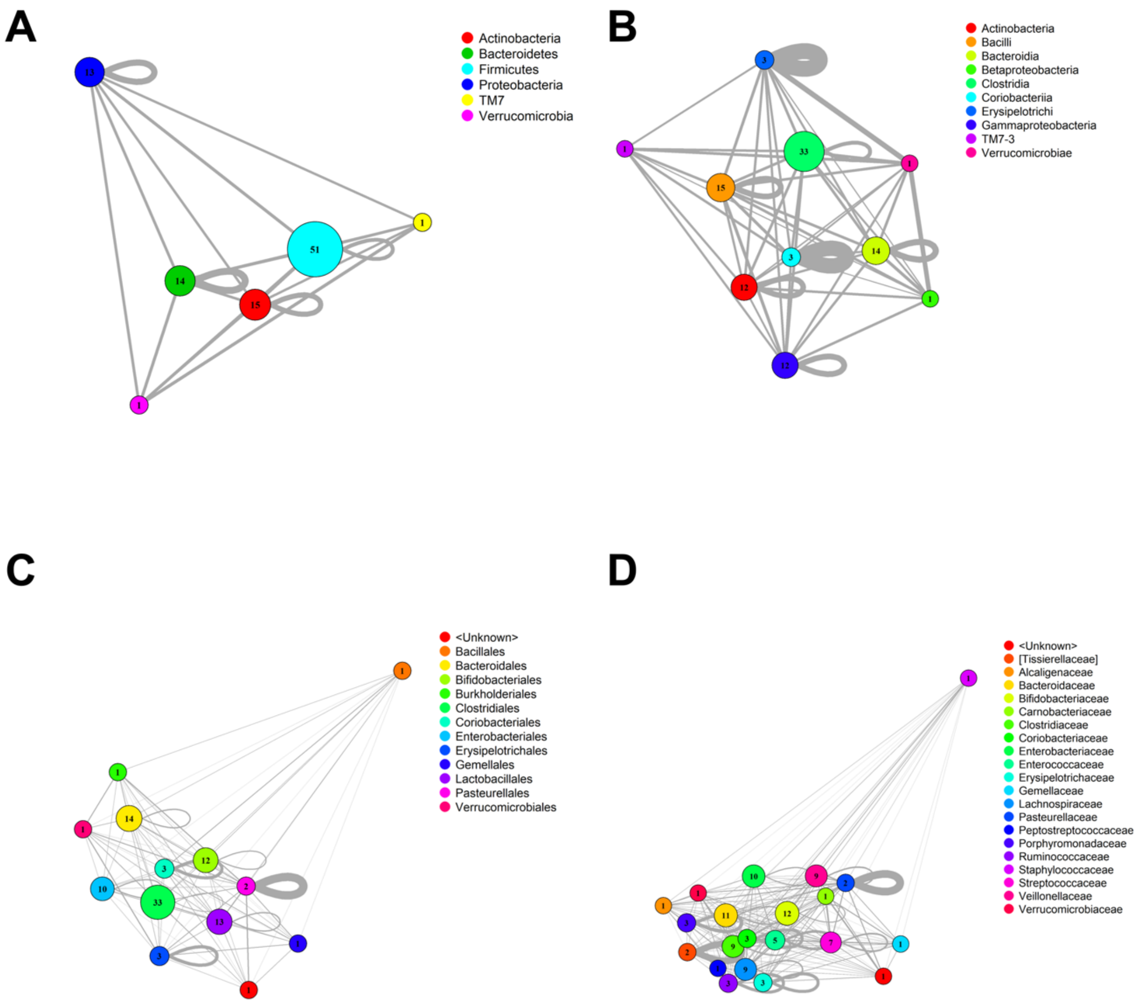

Figure S7: Representation of weighted taxa interactions at month 9, grouped for (A) Phylum, (B) Class, (C) and (D) Families with the SparCC method. SparCC does not downplay edges as much as MAGMA

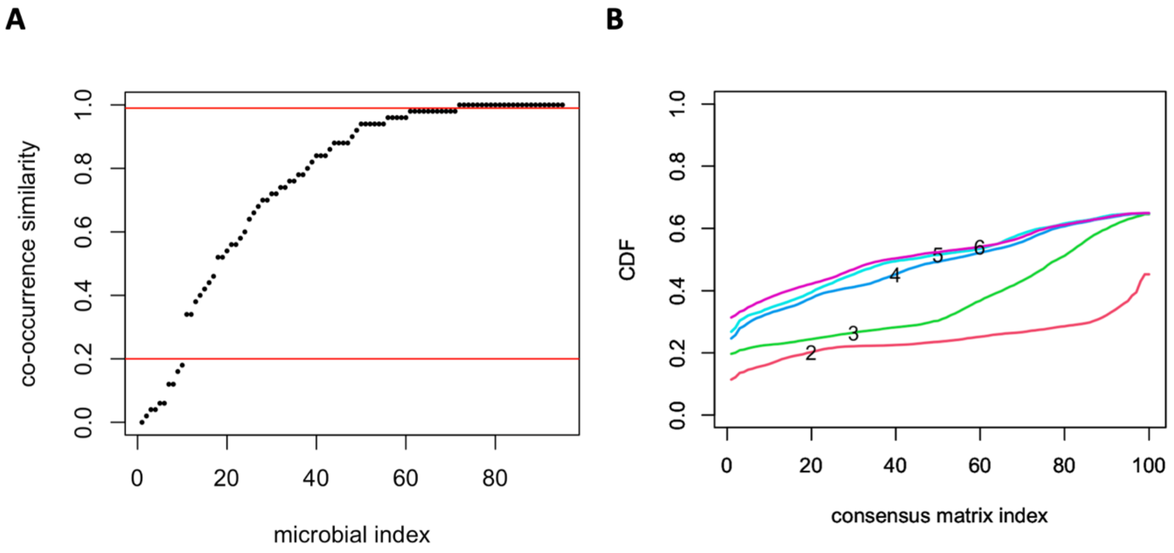

Figure S8: Assessing the microbial interaction variability. (A) The selection of the highly and lowly dynamical microbes based on co-clustering similarities for each node pair. (B) The result of consensus clustering commutive distribution suggesting two robust clusters of microbes.

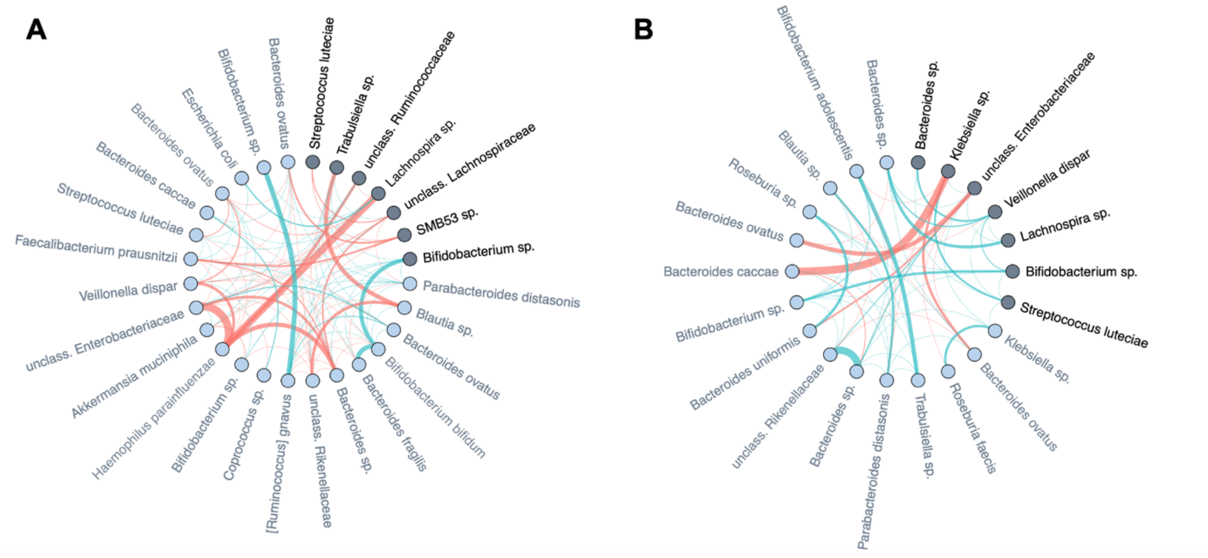

Figure S9: Differences of averaged microbial co-expression networks depicted in Figure 10. (A) the difference network of c-section delivery (Fig. 10A) subtracted from the difference network of vaginal delivery (Fig. 10B); (B) the difference network of non-persistent diet (Fig. 10C) subtracted from the difference network of persistent diet (Fig. 10D).

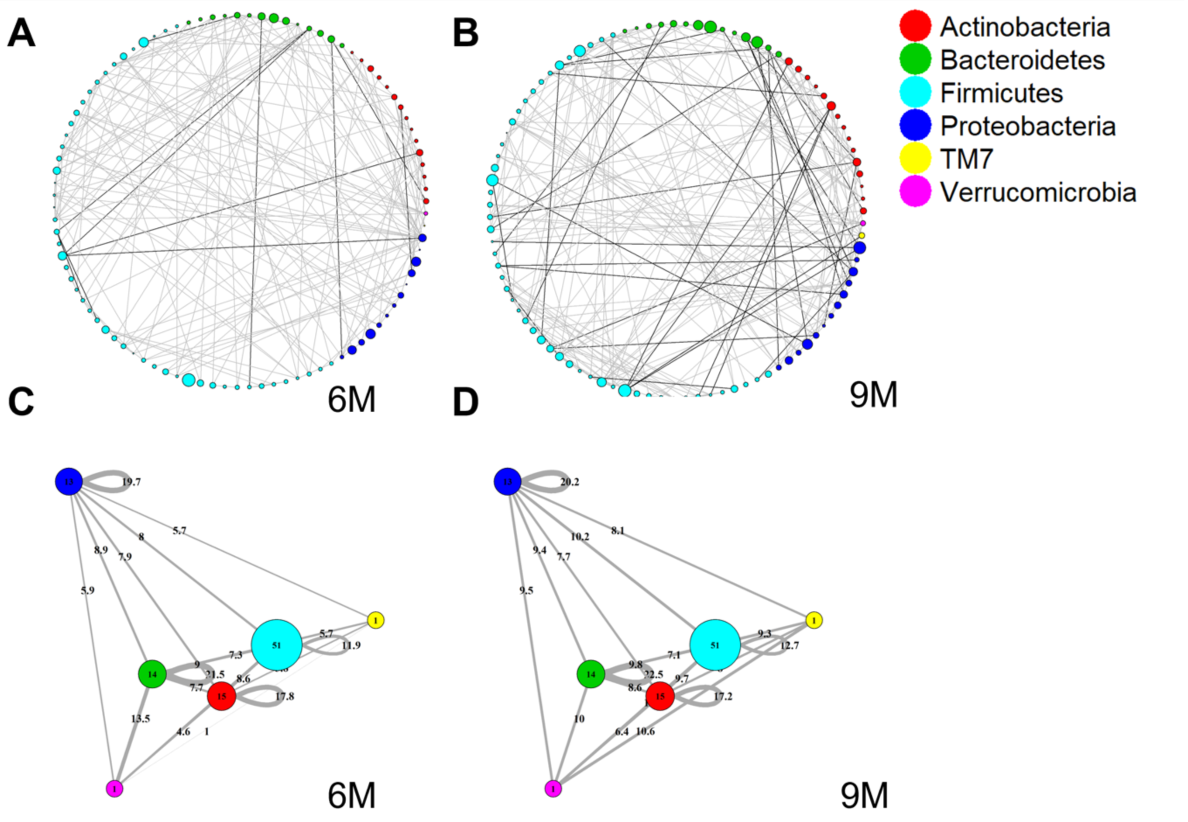

Figure S10: Global SparCC networks calculated on all subjects retained in the LucKi cohort at 6 (81) and 9 (74) months after birth. Colour code corresponds to phylum classification. The thickness of an edge corresponds to the strength of the association. Microbial taxa are organized in a circle according to phylum membership at 6m (A) and 9m (B). In (C) and (D), respectively, time points 6m and 9m, edges are aggregated per phylum. Within- and across- phyla co-occurrences are computed via SparCC continuous networks at each time point. The nodes' and edges' thickness correspond to phylum size and association strength.

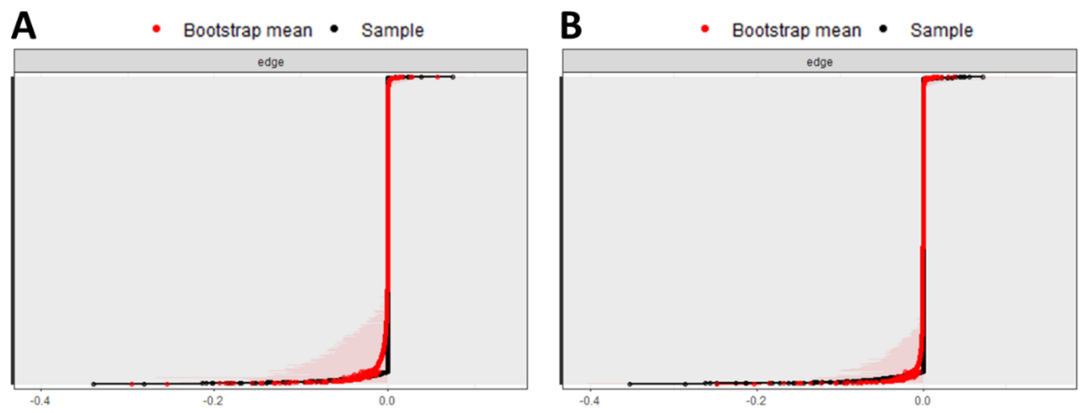

Figure S11: Edge weight stability. Mean edge weight across bootstrap replicates with 95% confidence interval are shown. X-axis: edge weight; Y-axis: edge annotation. Edges are ordered according to the inferred MAGMA weights in the original sample.

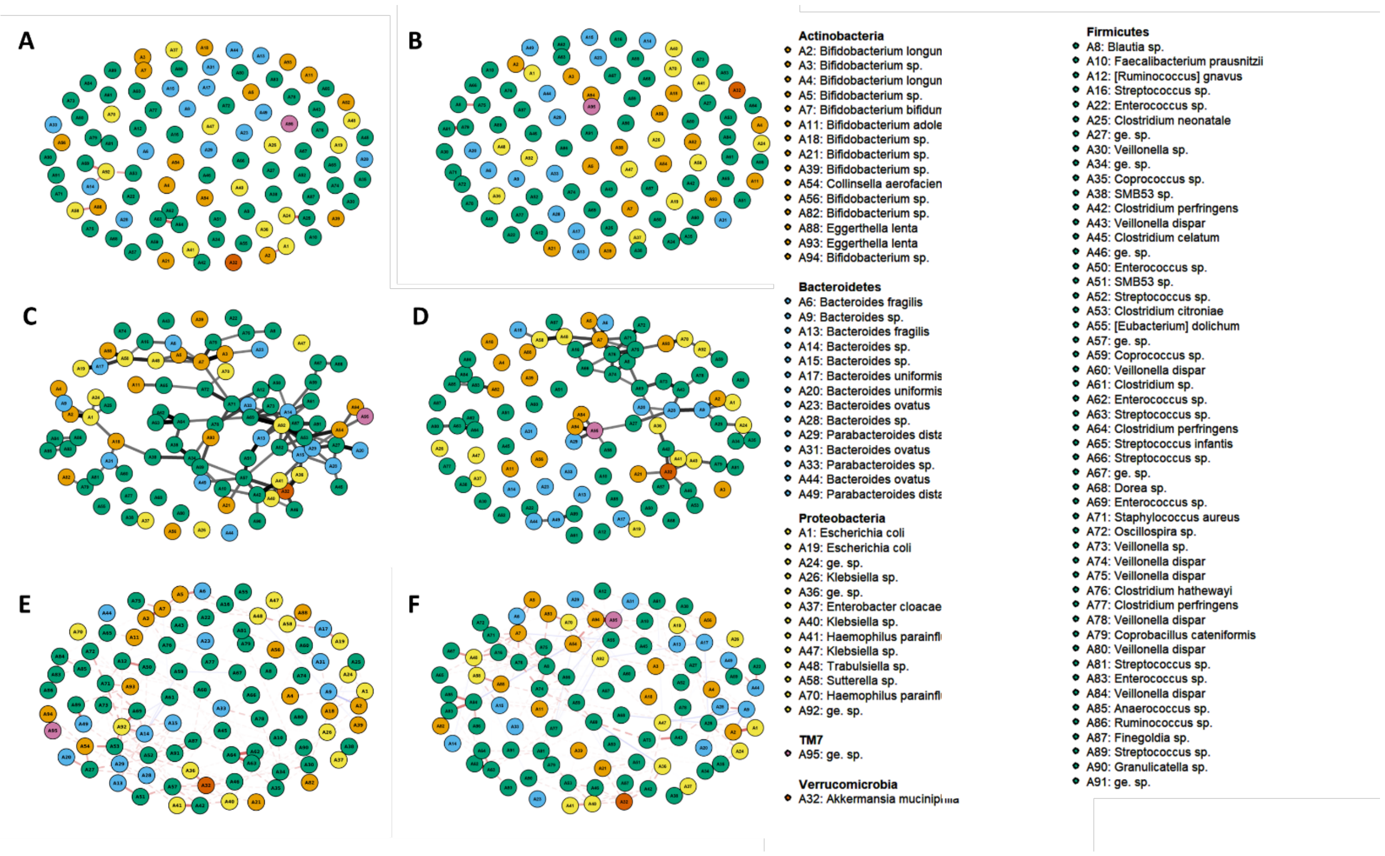

Figure S12: Threshold MAGMA bootstrap networks and inclusion MAGMA networks, as explained in the text. Networks with bootstrap significant edges (α = 5%): A-6m, 12 edges; B-9m, 8 edges). Inclusion network with edges present in more than half of the bootstrap replicates (>500): C-6m, 118 edges; D-6m, 82 edges. For reference, the discovery MAGMA networks for 6m (E, 184 edges) and 6m (F, 216 edges) are also shown. Nodes are colour-coded based on the Phyla, and enumerated (*A1,A2*, ..) with their Genus-Species.

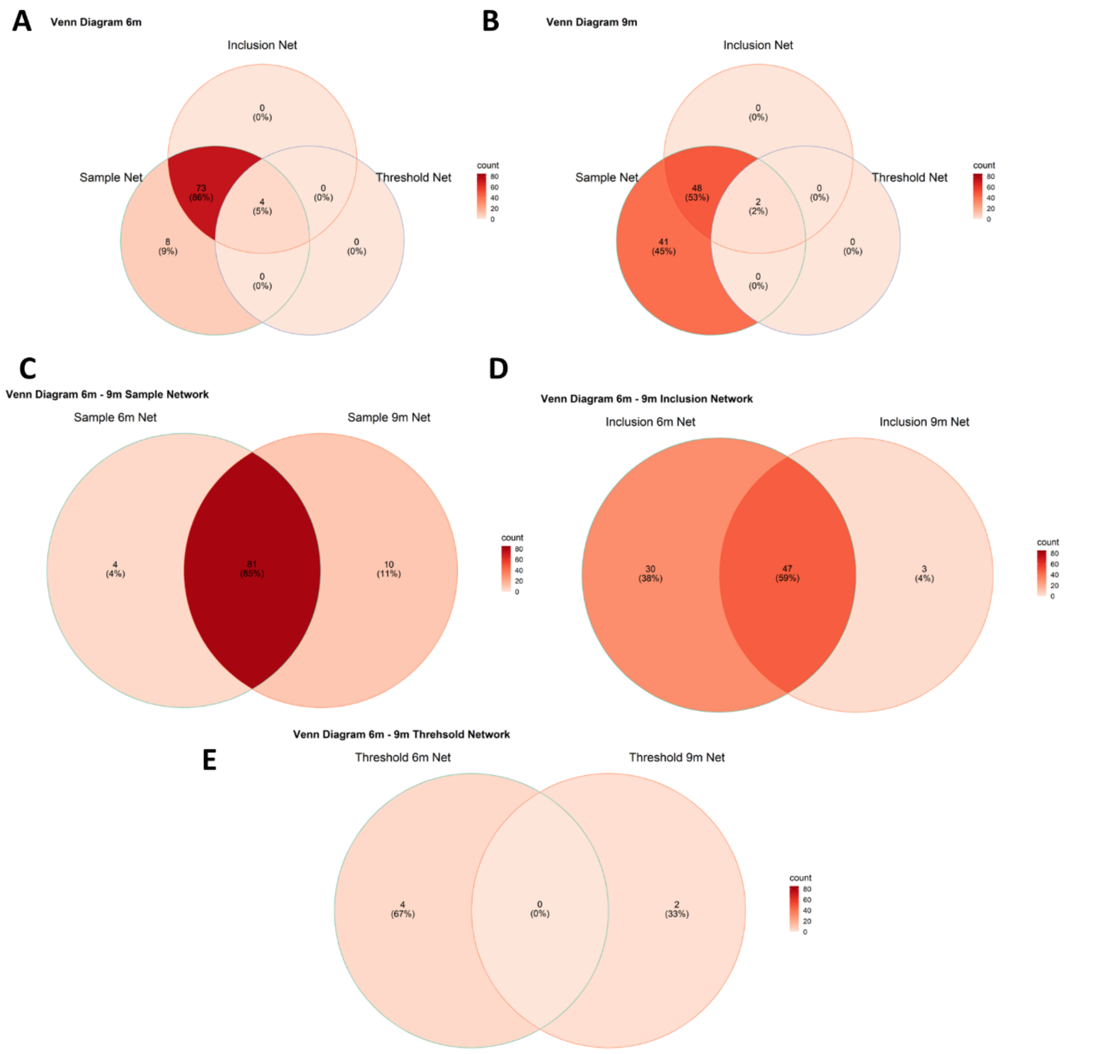

Figure S13: Venn diagram for the nodes in the giant component (details in text). In (A) and (B), the intersection between the discovery network (calculated on the original data), bootstrap inclusion and threshold network, at 6m and 9m, respectively. In (C), (D) and (E), the intersection between the nodes in the giant component between, respectively, the sample network at 6m and 9m; the inclusion network at 6m and 9m and the threshold network (6m and 9m).

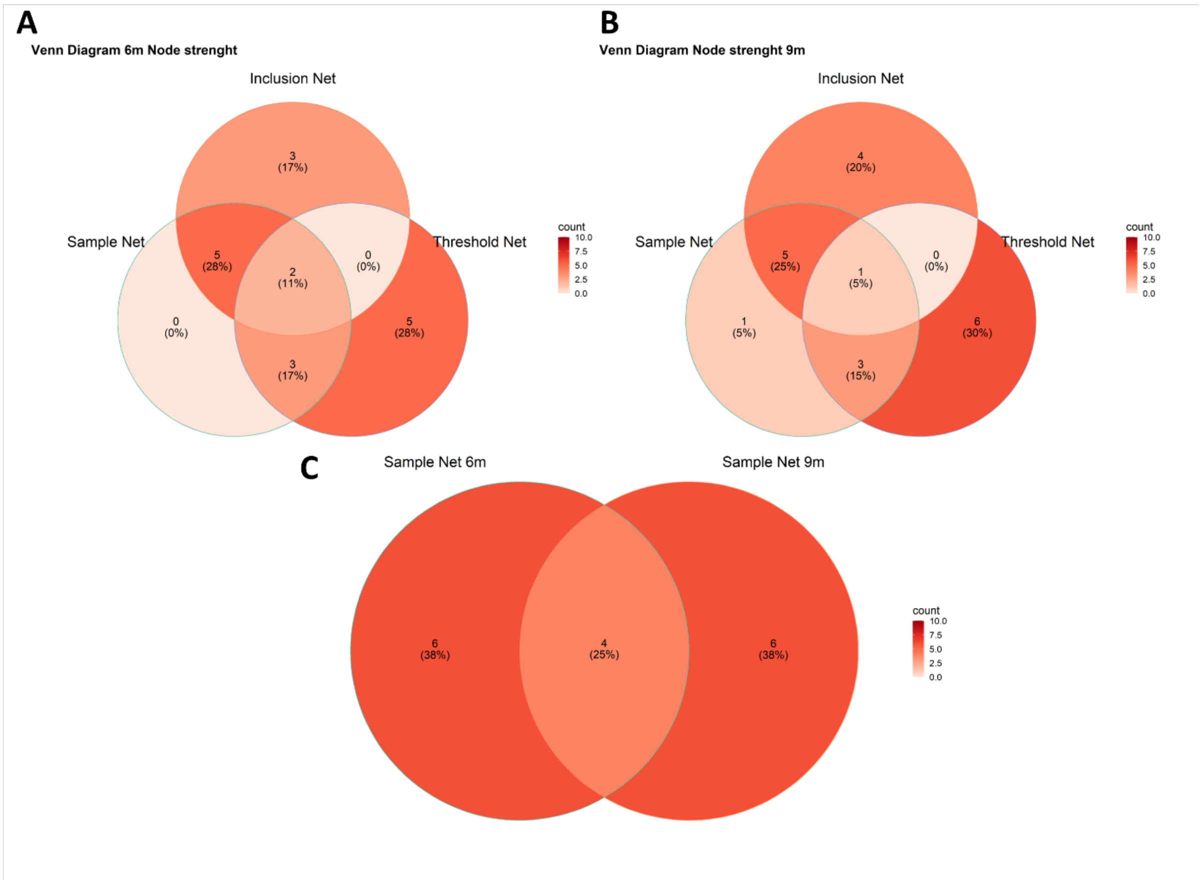

Figure S14: Venn Diagram for the top-10 nodes for node strength. In (A) and (B), the intersection of the nodes with top-10 node strength between the sample network (calculated on the original data), inclusion MAGMA network, and threshold MAGMA bootstrap network, respectively, at 6m and 9m. In (C), we show the intersection between nodes in the top 10 in the sample network calculated at 6m and 9m.

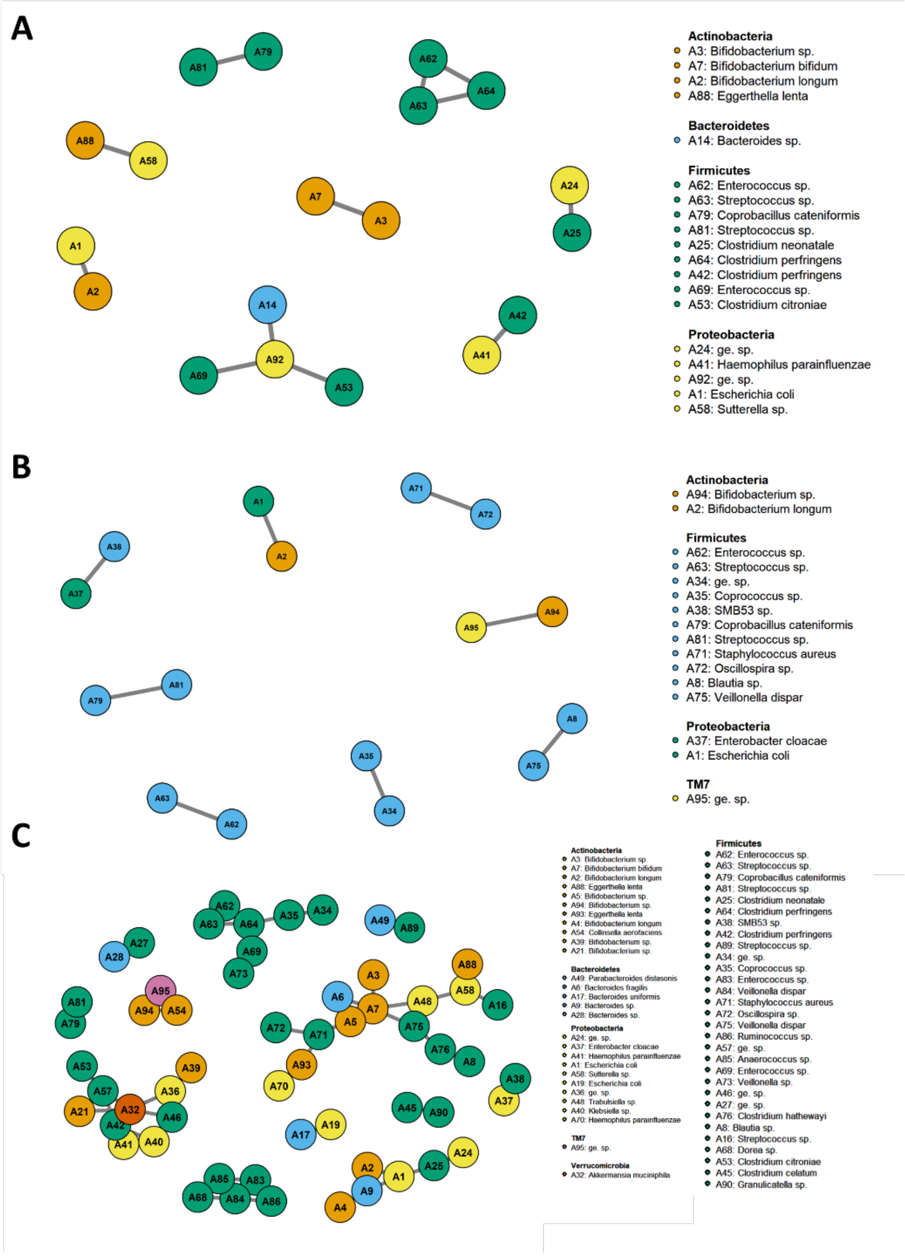

Figure S15: Intersection of edges in MAGMA threshold, and inclusion bootstrap networks and the original discovery networks. In (A), the intersection of edges presents in threshold network, inclusion network and discovery network at 6m, with 12 edges; in (B) at 9m, with 8 edges. For completion in (C), we show the intersection of discovery networks, in particular edges at 6m and 9m: 59 edges. Nodes are colour-coded based on the *Phyla*, and enumerated (*A1,A2*, ..) with their *Genus-Species.*

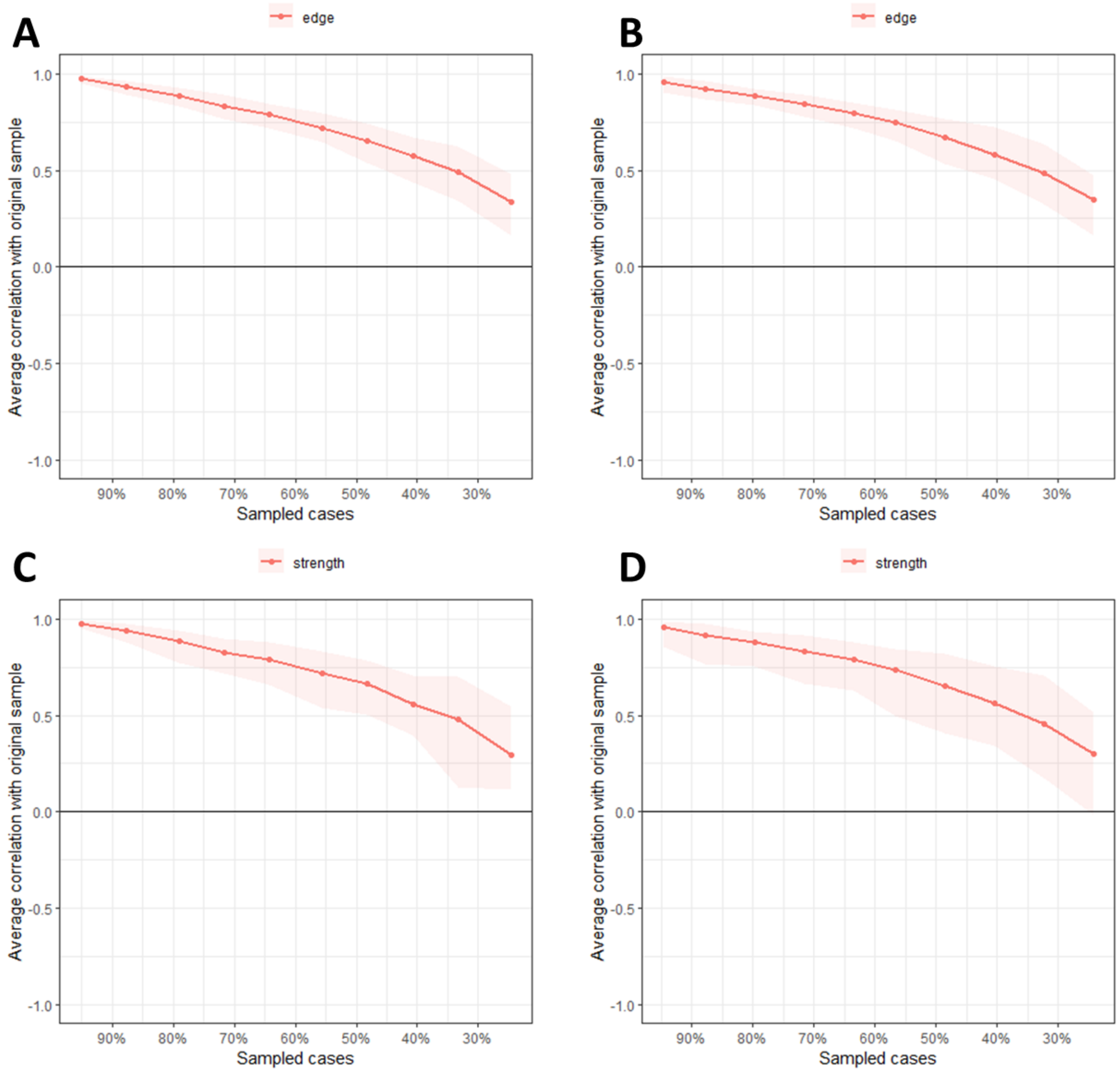

Figure S16: Average Correlation coefficient (CS) between case-dropping bootstrap replicates and original dataset for edge weight (A: 6m; B: 9m). For information, the average CS for node strength is also shown (C: 6m; D: 9m). In particular, for each percentage of samples left out from the original data, CS was computed for each edge (each node in case of node strength) and was averaged across edges (nodes). Variation across bootstrap replicates is shown via coloured areas, referring to 95% bootstrap intervals.

Table SI. List of microbes with dissimilar local neighborhood dynamics between m6 and m9. Genus-species names are shown. See main text for the definitions of the clusters.

	**Amplicon sequence variants (ASVs)**
**Cluster 1 to cluster 2**	*Akkermansia muciniphila, Bacteroides fragilis, Bacteroides ovatus, Bifidobacterium sp., Bifidobacterium sp., Clostridium perfringens, Collinsella aerofaciens, Faecalibacterium prausnitzii, SMB53 sp., Streptococcus luteciae, Streptococcus sp., Streptococcus sp.*
**Cluster 2 to cluster 1**	*Bacteroides ovatus, Bacteroides sp., Bacteroides sp., Bifidobacterium adolescentis, Bifidobacterium longum, Enterobacteriaceae unclassified, Enterococcus sp., Escherichia coli, Faecalibacterium prausnitzii, Parabacteroides sp., Ruminococcus bromii, Ruminococcus gnavus*

Table SII: Kendall's *tau* test p-value for the ranking of node strength between thresholded MAGMA bootstrap networks, inclusion MAGMA networks and the original sample network.

Network		Threshold network		Inclusion network		Sample network	
	Time point	6m	9m	6m	9m	6m	9m
**Threshold network**	6m	/	0.0292	4.829e-04	/	1.440e-08	/
	9m	0.0292	/	/	4.393e-04	/	1.799e-06
**Inclusion network**	6m	4.829e-04	/	/	1.044e-03	< 2.2e-16	/
	9m	/	4.393e-04	1.044e-03	/	/	< 2.2e-16
**Sample network**	6m	1.440e-08	/	< 2.2e-16	/	/	7.005e-04
	9m	/	1.799e-06	/	< 2.2e-16	7.005e-04	/

Table SIII: Kendall's *tau* test values for the ranking of node strength between thresholded MAGMA bootstrap networks, inclusion MAGMA networks and the original sample network. The *tau* value is bounded between -1 and 1 and indicates the degree of agreement between the two ranking.

Network		Threshold network		Inclusion network		Sample network	
	Time point	6m	9m	6m	9m	6m	9m
**Threshold network**	6m	/	0.207	0.284	/	0.459	/
	9m	0.207	/	/	0.296	/	0.390
**Inclusion network**	6m	0.284	/	/	0.237	0.750	/
	9m	/	0.296	0.237	/	/	0.780
**Sample network**	6m	0.459	/	0.750	/	/	0.237
	9m	/	0.3899861	/	0.7800079	0.236668	/

References:

Cougoul, A., Bailly, X., & Wit, E. C. (2019). MAGMA: inference of sparse microbial association networks. *BioRxiv*, 538579. https://doi.org/10.1101/538579

Friedman, J., & Alm, E. J. (2012). Inferring Correlation Networks from Genomic Survey Data. *PLoS Comput Biol*, *8*(9), 1002687. https://doi.org/10.1371/journal.pcbi.1002687

Gosak, M., Markovič, R., Dolenšek, J., Slak Rupnik, M., Marhl, M., Stožer, A., & Perc, M. (2018). Network science of biological systems at different scales: A review. *Physics of Life Reviews*, *24*, 118–135. https://doi.org/10.1016/J.PLREV.2017.11.003

Jahagirdar, S., & Saccenti, E. (2020). Evaluation of Single Sample Network Inference Methods for Metabolomics-Based Systems Medicine. *Journal of Proteome Research*, *20*(1), 932–949. https://doi.org/10.1021/ACS.JPROTEOME.0C00696

Kishore, D., Birzu, G., Hu, Z., DeLisi, C., Korolev, K. S., & Segrè, D. (2020). *Inferring microbial co-occurrence networks from amplicon data: A systematic evaluation* (p. 2020.09.23.309781). bioRxiv. https://doi.org/10.1101/2020.09.23.309781

Kuijjer, M. L., Hsieh, P.-H., Quackenbush, J., & Glass, K. (2019). LionessR: Single sample network inference in R. *BMC Cancer*, *19*(1). https://doi.org/10.1186/s12885-019-6235-7

Kuijjer, M. L., Tung, M. G., Yuan, G., Quackenbush, J., & Glass, K. (2019). Estimating Sample-Specific Regulatory Networks. *IScience*, *14*, 226–240. https://doi.org/10.1016/j.isci.2019.03.021

Liu, X., Wang, Y., Ji, H., Aihara, K., & Chen, L. (2016). Personalized characterization of diseases using sample-specific networks. *Nucleic Acids Research*, *44*(22), e164–e164. https://doi.org/10.1093/NAR/GKW772

Monti, S., Tamayo, P., Mesirov, J., & Golub, T. (2003). Consensus Clustering: A Resampling-Based Method for Class Discovery and Visualization of Gene Expression Microarray Data. *Machine Learning*, *52*(1), 91–118. https://doi.org/10.1023/A:1023949509487

Zhao, T., Liu, H., Roeder, K., Lafferty, J., & Wasserman, L. (2020). *The huge Package for High-dimensional Undirected Graph Estimation in R* (arXiv:2006.14781). arXiv. http://arxiv.org/abs/2006.14781
